# Update on the Medical Management of Gastrointestinal Behçet's Disease

**DOI:** 10.1155/2017/1460491

**Published:** 2017-01-22

**Authors:** Giuseppe Lopalco, Donato Rigante, Vincenzo Venerito, Claudia Fabiani, Rossella Franceschini, Michele Barone, Giovanni Lapadula, Mauro Galeazzi, Bruno Frediani, Florenzo Iannone, Luca Cantarini

**Affiliations:** ^1^Department of Emergency and Organ Transplantation, Rheumatology Unit, University of Bari, Piazza Giulio Cesare 11, 70124 Bari, Italy; ^2^Institute of Pediatrics, Università Cattolica del Sacro Cuore, Fondazione Policlinico Universitario A. Gemelli, Largo A. Gemelli 8, 00168 Rome, Italy; ^3^Department of Ophthalmology, Humanitas Clinical and Research Center, Via Manzoni 56, Rozzano, 20089 Milan, Italy; ^4^Ophthalmology and Neurosurgery Department, University of Siena, Viale Bracci 1, 53100 Siena, Italy; ^5^Section of Gastroenterology, Department of Emergency and Organ Transplantation, University of Bari, Piazza Giulio Cesare 11, 70124 Bari, Italy; ^6^Research Center of Systemic Autoinflammatory Diseases and Behçet's Disease Clinic, Department of Medical Sciences, Surgery and Neurosciences, University of Siena, Viale Bracci 1, 53100 Siena, Italy

## Abstract

Behçet's disease (BD) is a multisystemic disorder of unknown etiology mainly defined by recurrent oral aphthosis, genital ulcers, and chronic relapsing bilateral uveitis, all of which represent the “stigmata” of disease. However, many other organs including the vascular, neurological, musculoskeletal, and gastrointestinal systems can be affected. The gastrointestinal involvement in Behçet's disease (GIBD), along with the neurological and vascular ones, represents the most feared clinical manifestation of BD and shares many symptoms with inflammatory bowel diseases, such as Crohn's disease and ulcerative colitis. Consequently, the differential diagnosis is often a daunting task, albeit the presence of typical endoscopic and pathologic findings may be a valuable aid to the exact diagnosis. To date, there are no standardized medical treatments for GIBD; therefore therapy should be tailored to the single patient and based on the severity of the clinical features and their complications. This work provides a digest of all current experience and evidence about pharmacological agents suggested by the medical literature as having a potential role for managing the dreadful features of GIBD.

## 1. Introduction

Behçet's disease (BD) is a rare relapsing systemic inflammatory disorder of unknown etiology characterized by recurrent oral ulcers, genital sores, and ocular lesions; however many other organs including the vascular, neurological, and musculoskeletal systems as well as the gastrointestinal system can be involved [1–3]. Genetic and environmental factors play a key role in this disorder, in particular the human leukocyte antigen B51 allele, located in the major histocompatibility complex locus, representing the strongest risk factor for the development of BD [4]. In recent years, some microbial agents such as Herpes simplex virus 1 and* Streptococcus sanguinis* have gained increasing importance as potential infectious agents of BD [5], being able to generate an inflammatory process leading to a CD4+ T lymphocytes clonal expansion which in turn produces high concentrations of both proinflammatory cytokines and cytotoxic CD8+ cells [6]. Several cytokines are claimed to contribute to the pathological scenario of BD [5, 7–9]: tumor necrosis factor- (TNF-) *α* partakes probably in somehow the disease onset and the successful use of anti-TNF-*α* agents has substantiated the role of this cytokine in BD [10–12]. Conversely interleukin- (IL-) 6 seems to be related to central nervous system involvement, as confirmed by its high levels in the cerebral spinal fluid of affected patients [13]. Recent studies have also suggested a role of IL-1, since its secretion in BD patients appeared to be related to NLRP3 inflammasome activation [14–17].

Although oral aphthae and genital ulcers are the earliest and the most frequent manifestations of BD, anticipating by many years other typical BD clinical symptoms, GIBD is one of the major causes of morbidity and mortality, often leading to severe complications. GIBD occurs in 3–60% of patients, on average 4.5–6 years after the onset of oral ulcerations [18], varying among different populations [2, 19–21] and being more frequent in Japan, United Kingdom, and Taiwan than in the Middle East and Mediterranean basin [4, 22, 23]. Intestinal ulcerations are the main pathological features of GIBD, and it is thought they are secondary to small vessel vasculitis, albeit a large vessel involvement leading to ischemic damage may arise [24]. GIBD may be suspected when diarrhoea, melena, and hematochezia occur [25–27]. Common complaints may include abdominal pain, fever, anorexia, vomiting, and dyspepsia, and a palpable mass on the affected quadrant can also be noticed [25]. The terminal ileum is the most common localization of disease, followed by the ileocecal region and colon [28]; esophagus engagement is unusual [29], and rectum or anus is also rarely affected [30] while the stomach is the least frequently involved part of the gastrointestinal tract [31, 32].  Table 1 shows the most common intestinal localizations of BD.

Gastrointestinal lesions are typically irregular, round or oval, punched-out, large (>1 cm), single to a few in number, deep, and with discrete margins in a focal distribution [33]. On the basis of endoscopic findings, they are classified into volcano, geographic, and aphthous types. The volcano-type, deeply penetrating and having nodular margins caused by fibrosis, is strictly associated with a poor prognosis [34, 35]. The differential diagnosis between GIBD and inflammatory bowel disease, in particular Crohn's disease (CD), is often difficult, albeit in the latter the ulcers have typically a cobblestone appearance with a segmental distribution which involves irregularly different parts of the gastrointestinal tract [33]. In this regard a diagnostic algorithm using a classification analysis of the lesions has been proposed in order to identify valuable strategies for differential diagnosis [36]. A clinical scoring system known as the Disease Activity Index for Intestinal BD (DAIBD) provides a score between 0 and 325 based on an 8-point index; it classifies disease activity as quiescent (≤19), mild (20–39), moderate (40–74), and severe (≥75) on the basis of patient's general condition, extraintestinal manifestations, intestinal symptoms and signs, and stool frequency [37].  Table 1 summarizes the main clinical, endoscopic, and pathological findings of GIBD.

Although a wide number of conventional immunosuppressive drugs have been used to induce remission in GIBD, several failures have been reported. This article reviews the progress in the management of GIBD focusing on current treatment strategies and possible future perspectives. An electronic literature search was conducted using the PubMed database and the clinicaltrials.gov search engine. We looked for all studies published in the last years, including case reports, clinical trials, and cohort studies (Table 2).

## 2. The Management of GIBD

As underlined in the guidelines of the European League Against Rheumatism (EULAR) for the management of BD, evidence-based recommendations regarding GIBD are not provided due to the poor amount of published clinical trials [38]. Medical treatment such as corticosteroids (CC), sulfasalazine (SSZ), and azathioprine (AZA) are capable of inducing remission without the need for surgery in many patients [25, 39], whereas TNF-*α* antagonists and thalidomide (THD) have proven useful in resistant and complicated cases [10–12, 40, 41].

Nevertheless GIBD management is still largely empirical due to the lack of not yet standardized medical treatments, the heterogeneity of this disorder, and the unpredictable exacerbations of BD. To date, several conventional immunosuppressive drugs may be employed, although none of them has been proven actually effective in preventing disease relapse.

### 2.1. Corticosteroids (CC)

CC are the first-line therapy, especially in patients with severe systemic symptoms, recurrent gastrointestinal bleeding, or when treatment with 5-aminosalicylic acid (5-ASA)/SSZ is not enough [42]. CC are supposed to be very effective in the short term; indeed it is widely accepted to start with prednisolone or its equivalent at 0.5–1 mg/kg which has to be quickly tapered by 5 mg each week within few months [43]. The rationale behind this strategy may be figured out noticing the evidence reported by Park et al. in a retrospective cohort study; systemic CC therapy (mean starting dose, 0.58 mg/kg) was administered in 54 patients with active GIBD; a complete remission and partial remission were achieved in 46.3% and 42.6% of patients respectively, whereas only 11.1% showed no response one month after starting treatment. At one-year follow-up, a prolonged response was found in 26 out of 54 patients, whereas 19 patients showed CC dependency, suggesting that their employment is not desirable over extended periods [44]. Several literature data report the association between CC employment and GI side effects, including bleeding or perforation. GI bleeding and perforation are assumed to occur when ulcers erode into underlying vessels. CC may impair tissue repair, thus leading to delayed wound healing. Despite these assumptions, a recent systematic review and meta-analysis of randomised, double-blind, controlled trials comparing CC to placebo for any medical condition or in healthy participants have suggested that additional factors to corticosteroid therapy, such as severe physiological stress, may decrease mucosal blood flow with subsequent tissue ischemia making some patients more vulnerable to adverse events under CC assumption. Therefore acid-suppressive therapy may be considered a valuable aid in preventing the occurrence of ulcers in clinical settings [45].

### 2.2. 5-Aminosalicylic Acid (5-ASA)/Sulfasalazine (SSZ)

5-ASA/SSZ is indicated in all cases of GIBD due to its safety profile and current limited therapeutic options [46]. It is usually administered at a dose of 2–4 g/day for inducing remission in mild forms of BD and for maintenance once remission is achieved [27]. Convincing evidence about the efficacy of 5-ASA derives from nonrandomized studies and case series suggesting that it is effective in treating esophageal and gastrointestinal manifestations of BD [47, 48], albeit conflicting data regarding its usefulness have been reported as well [49]. In a retrospective cohort study investigating 143 patients with GIBD who received 5-ASA/SSZ alone for maintaining remission, cumulative relapse rates at 1, 3, 5, and 10 years after remission were 8.1%, 22.6%, 31.2%, and 46.7%, respectively. Of note, a younger age at diagnosis (<35 years), higher serum level of C-reactive protein (1.5 mg/dL), and greater DAIBD score (≥60) were regarded as independent predictors of relapse [50]. More recently an observational study has suggested 5-ASA as a valuable treatment for preventing postoperative recurrences; remission was achieved in 10 out of 16 (62.5%) patients who took 5-ASA compounds, and no exacerbation was seen during the 89.3 ± 64.5 months that they were followed. Similarly, remission was observed also among 37 patients who were prescribed with azathioprine, and there were no relapses in 24/37 (65%) patients during a mean follow-up of 68.6 ± 43.6 months [51].

### 2.3. Thalidomide (THD)

THD is an immunomodulatory drug, used mainly in the treatment of specific tumors. It is usually considered the last-line therapy for GIBD, albeit its use is well-documented [52]. The immunomodulatory effects of THD are due to the reduction in levels of TNF-*α* because of degradation of its encoding mRNA [53]. A pilot study [54] and three open studies [55–57] have demonstrated THD effectiveness in the treatment of BD with mucocutaneous involvement as well as in CD enteric involvement [58].

Yasui et al. reported the benefits of THD in 7 juvenile‐onset patients with severe, recurrent GIBD who had previously failed immunosuppressant treatments and developed significant CC toxicity. THD was given at the initial dose of 2 mg/kg per day and was increased to 3 mg/kg per day if necessary. All patients showed dramatic improvement in clinical symptoms, and CC were successfully withdrawn [59]. Yet, the efficacy of THD has been also reported on four BD patients with relapsing gastrointestinal disease who required the frequent use of systemic CC. Three out of the four patients had a clinical improvement on THD treatment and all discontinued CC therapy suggesting that THD could be considered a therapeutic option for treatment of refractory GIBD [60]. More recently Hatemi et al. described their experience on 13 patients with GIBD refractory to the conventional therapy who were treated with TNF-*α* antagonists and/or THD; a clinical and endoscopic remission was obtained in 10 out of 13 patients (about 75% of the cases) proving the favourable response with anti-TNF-*α* agents and/or THD in GIBD [61].

Despite the efficacy and safety profile reported in several bodies of evidence of the literature [59, 62, 63], more data from clinical trials are necessary to define the proper use of this widely known teratogenic drug.

### 2.4. Other Immunomodulators

Thiopurines including 6-mercaptopurine (6-MP) and its prodrug AZA have been traditionally thought to decrease reoperation rate in patients with GIBD who already had undergone surgical interventions [25]. The initial dose of AZA is 25–50 mg/day with gradual increase every 2–4 week to 2.0–2.5 mg/kg [64]. Similarly the starting dose of 6-MP is 0.5 mg/kg escalated every 2–4 weeks to an optimal dosing regimen of 1.0–1.5 mg/kg. Data on the questionable effectiveness of thiopurine treatment in patients with GIBD derive from a retrospective analysis aimed at investigating predictors of clinical relapse in 67 patients with GIBD receiving thiopurine maintenance therapy; a cumulative relapse rates of 5.8%, 28.7%, 43.7%, and 51.7% at 1, 2, 3, and 5 years, respectively, after remission were recorded. Although thiopurine therapy has proven to be relatively effective for maintenance of remission in GIBD, a younger age at diagnosis (<25 years) and a lower hemoglobin level (<11 g/dL) were associated with a poor response to this treatment [65].

Yet, a recent retrospective observational study was carried out to assess the efficacy of postoperative thiopurine therapy in 77 patients with GIBD; although a lower postoperative recurrence was found in patients who received thiopurines than those taking 5-ASA, the rates of reoperation, readmission, and death were not significantly different between the 5-ASA and thiopurine groups [66].

Evidence concerning the efficacy of methotrexate combined with infliximab (IFX) in refractory entero-BD has been also reported in 9 out of 10 patients who experienced the disappearance of ileocecal ulcerations at 12 months of therapy [67]. In addition, anecdotal case reports describing the effectiveness of tacrolimus [68] and chlorambucil [69] for treating intestinal lesions in BD have been also described.

Interferon (IFN) is a cytokine able to render cells resistant to infection by many viruses. It was introduced for the treatment of BD by Tsambaos in 1986, because of its antiviral and antiproliferative properties [70]. The most impressive results have been achieved for severe and/or refractory ocular manifestations; however IFN-*α* could represent a promising treatment option for neurologic and gastrointestinal involvement in BD [71]. In this regard Grimbacher et al. reported a complete remission of BD retinal infiltrates and BD-related colitis after treatment with IFN-*α* [72], and similar proofs supporting the benefits of IFN-*α* also derive from the study by Monastirli et al. [73].

During the last few decades intravenous immunoglobulins (IVIg) have been increasingly administered for a wide number of autoimmune and systemic inflammatory diseases. To the best of our knowledge, IVIg have so far been evaluated in few patients with GIBD. In this regard, the efficacy of IVIg has been reported in a patient with BD-related colitis who initially had failed under CC and immunosuppressive therapy [19], as well as in a patient with GIBD complicated by the presence of immune deficiency [74]. More recently, four BD patients, one of whom suffered from neurological and gastrointestinal involvement, refractory to standard treatments and responsive to IVIg therapy have also been described [75].

### 2.5. Biological Drugs

Several data suggest a role of TNF-*α* in the pathogenesis of BD; a remarkable upregulation of TNF-*α* and soluble TNF receptors [76] as well as a great amount of *γδ*+ T cells producing TNF were found in the peripheral blood of patients with active disease [77]. Currently, the monoclonal antibodies anti-TNF-*α* IFX [78] and adalimumab (ADA) [79] along with the human TNF receptor p75 Fc fusion protein etanercept (ETN) [80] have been advocated for the treatment of different BD manifestations. The administration schedule of IFX for treating GIBD is adopted from the regimen employed in the management of CD (5 mg/kg intravenous at weeks 0, 2, and 6) [81]. A clinical remission of intestinal BD lesions and the rapid healing of ulcers after treatment with IFX have been described in several case reports (10, 40, 82–90). The short and long-term effects of IFX on the clinical course and intestinal manifestations of BD were assessed by abdominal computed tomography and colonoscopy in ten patients with entero-BD refractory to the conventional therapies; all patients showed improvement of gastrointestinal symptoms and disease-associated complications within 4 weeks. Furthermore, the rate of disappearance of ileocecal ulcerations was 50% (5/10 patients) at 6 months and 90% (9/10 patients) at 12 months [67]. On the contrary, the results derived from a retrospective observational study enrolling 43 patients with GIBD were not entirely encouraging; in this context, IFX chosen as optional treatment in 7 patients refractory to conventional therapies led to clinical remission only in one case (14%) [82].

Yet, a Korean multicenter retrospective study aimed at investigating the response to IFX in 28 patients with GIBD showed a clinical response rate of 64.3% with a clinical remission rate of 28.6% at week 4, following IFX infusion. Furthermore, an older age at diagnosis (≥40 years), the female sex, a longer disease duration (≥5 years) as well as the concomitant immunomodulator use, and achievement of remission at week 4 were regarded as predictive factors of sustained response [83].

The efficacy of IFX in GIBD has also been corroborated by a retrospective cohort study on 15 patients with active disease refractory to conventional medications. 80% of patients exhibited a good response to IFX and 53% of them achieved remission after 10 weeks. Moreover 64% and 50% of patients maintained the response to IFX at 12 and 24 months, respectively [84]. More recently an open-label single-arm phase 3 study carried out on 18 BD patients including 11 with GIBD suggested that IFX was able to induce a clinical amelioration along with decrease in C-reactive protein levels after week 2. Consistently, the healing of the main intestinal ulcers was found in more than 80% of these patients after week 14. Interestingly, 3 patients who had loss of response to IFX showed complete resolution of symptoms by increasing its dosage to 10 mg/kg [85].

Finally, an interventional open-label single-arm study testing the efficacy of IFX by assessing the mean decrease in DAIBD score in patients with active intestinal disease refractory to conventional therapies is currently recruiting participants (ClinicalTrials.gov, NCT02505568).

To date, few data are available regarding ADA efficacy in GIBD, although the proofs of its usefulness are increasing [86–88]. In this regard, ADA has been already successfully used in three patients with BD-related colitis/esophageal ulcers [89] and for the first time in the context of a familial case of GIBD [90].

However, the most consistent evidence regarding ADA efficacy derives from a phase 3, multicenter, open-label uncontrolled study evaluating Japanese patients with active intestinal BD nonresponsive to standard therapies. Twenty patients received induction treatment with ADA (160 mg at week 0, baseline, and 80 mg at week 2, followed by 40 mg every other week for 52 weeks); a composite index, combining GI symptoms and endoscopic assessments, was used to evaluate the efficacy of treatment. A marked improvement, defined as values ≤1 for both the global GI symptoms and endoscopic assessment scores, was seen in 60% of patients at week 52. Interestingly 20% of patients achieved a complete remission, defined as global GI symptoms and endoscopic scores of 0, at weeks 24 and 52, suggesting that ADA was an effective therapy to induce and maintain clinical improvement and remission in patients with GIBD [91]. More recently, a retrospective study on 8 BD patients with intestinal BD, confirmed the long-term (52 weeks) efficacy and safety of ADA for the treatment of GIBD [92]. Of note, two prospective observational studies (clinicaltrials.gov NCT02687828 and NCT01960790) testing the safety and efficacy of ADA for the treatment of GIBD are now ongoing.

Less experience has been gained focusing on the management of GIBD with ETA treatment [93]. Recently Ma et al. have proven the superiority of ETA in GIBD as compared to conventional therapy, assessing the disappearance of intestinal ulcers confirmed by endoscopy. The healing rate of intestinal ulcers in the group treated with ETA (19 patients) was 89.47%, whereas in the group undergoing conventional treatments (35 patients) it was 51.42%. Therefore, these results proved a better curative effect of ETA as compared to conventional therapies [94].

Although the employment of anti-IL-1 agents on various BD manifestations has been well-documented [95–97], limited data are available for their efficacy in GIBD, being represented only by single-case reports and small case series that describe a clinical amelioration of symptoms without a clear improvement of organic lesions [15–17]. Similarly, albeit IL-6 could be a relevant therapeutic target for refractory BD and its activity can be blocked using the anti-IL-6 receptor antibody tocilizumab [98, 99], inconsistent are the literature data concerning the efficacy of this biologic drug in managing BD clinical manifestations that differ from the neurological ones [100–102].

### 2.6. Stem Cell Transplantation

Haematopoietic stem cell transplantation (HSCT) has been used in the treatment of severe autoimmune and inflammatory conditions unresponsive to conventional therapy. Although several treatment options, including biologic agents, are till now available for BD management, there is still an unmet need for more effective therapies for patients who are refractory to conventional treatments. In this regard encouraging results derive from several case reports describing HSCT in GIBD patients transplanted for accompanying haematological conditions. A complete remission of GI findings was observed after HSCT and there was no need to treat patients with any medications [103, 104]. This evidence suggests that HSCT may be an effective alternative in BD patients with severe organ involvement, especially GI involvement refractory to immunosuppressives. However, one must make sure that the benefit outweighs the risks when developing a management strategy for these patients. Since HSCT can be a life-threatening procedure, mostly autologous transplants should be preferred to those allogenic which may lead to major complications such as infections, GVHD, and hepatic, renal, and pulmonary damage [105].

### 2.7. Surgery

Surgery is indicated when patients with GIBD are refractory to medical treatment or serious complications, such as when gastrointestinal bleeding, perforation, fistulae, obstructions, and abdominal masses occur [106]. Bowel perforation is one of the most feared complications of GIBD. In this regard a retrospective study analysing free bowel wall perforation in 129 subjects with GIBD showed that 25.6% of patients experienced surgery for bowel perforation. Of them, 42.4% showed postoperative recurrence and 33.3% underwent reoperation. In addition, a younger age at diagnosis (≤25 years), an experience of prior laparotomy, and the presence of volcano-shaped ulcers were regarded as independent risk factors for free bowel perforation [107]. Resection involving sufficient margin and including normal bowel has been widely accepted in surgery [108, 109]. However, some bodies of evidence have proven that resection length is not related to postoperative recurrence in patients with GIBD, prospecting a less invasive surgical approach [39, 106]. Intestinal leakage, perforation, and fistula formation seems to occur more frequently at the anastomotic site; consequently, the creation of a stoma is preferred over primary anastomosis [18].

## 3. Conclusions

BD is a complex syndrome characterized by significant heterogeneity of clinical manifestations with usually frequent relapses. GIBD is one of the most severe manifestations of this disease, causing serious complications such as perforation and gastrointestinal bleeding. GIBD shares many clinical features with inflammatory bowel diseases, and for this reason it represents a pitfall for physicians regarding differential diagnosis at the first presentation. Nevertheless, a careful evaluation of endoscopic findings may help in the diagnostic interpretation, whereas the endoscopic biopsy is necessary to confirm a histopathologic diagnosis. Treatments have been largely unsatisfactory, creating significant unmet needs, and the lack of evidence-based treatment makes the management of GIBD very challenging. Currently, several immunosuppressive drugs such as SSZ, CC, and AZA are generally well-tolerated but often associated with increased risk of disease relapses as a result of which surgery is required in many patients. In the last years, new pathogenetic hypotheses supported the use of biotechnological drugs, mostly TNF-*α* inhibitors, which represent new tools in the therapeutic armamentarium for managing patients with GIBD, particularly those who are resistant to conventional immunosuppressant drugs. On this basis the main goal of treatment should be aimed at avoiding and preventing the feared complications of GIBD that endanger the life of these patients.

## Figures and Tables

**Table 1:** Main clinical, endoscopic, and pathological features of gastrointestinal involvement in Behçet's disease and most common localization.

	Behçet's disease	Crohn's disease	Ulcerative colitis
Gastrointestinal manifestations	Anorexia, vomiting, dyspepsia, diarrhoea, abdominal pain, melena, hematochezia, fever	Anorexia, vomiting, dyspepsia, diarrhea, gastrointestinal bleeding, abdominal pain, fever	Rectal bleeding, diarrhoea, tenesmus, abdominal pain, hematochezia, fever

Pathological features	Vasculitis of the small veins and venules with deep ulcerations, generally without granulomas or cobblestoning, ischemic perforation, thrombosis	Transmural mucosal inflammation, inflammatory cell infiltrate (lymphocytes, plasma cells) with focal crypt irregularity and independent granulomas	Distortion of crypt architecture, crypt abscesses, lamina propria cellular infiltration (plasma cells, eosinophils, lymphocytes), shortening of the crypts, mucin depletion, lymphoid aggregates, erosions or ulcerations

Endoscopic findings	Round or oval ulcers, punched-out lesions with discrete margins (>1 cm), focal distribution (<5 ulcers)	Longitudinal ulcers, cobblestone appearance, aphthous ulcers showing longitudinal array	Mucosal erythema, fine granularity, loss of vascular marking, erosions, ulcers, spontaneous bleeding, luminal narrowing with pseudopolyps

Localization	Terminal ileum, ileocecal region, colon	Small bowel, upper-gastrointestinal tract	Starts in the rectum and extends proximally in a continuous manner through the entire colon

**Table 2:** Overview of studies derived from the medical literature reporting treatment indications of gastrointestinal lesions in Behçet's disease.

Drugs	Dose	Authors (year)	Number of patients	Type of study	Outcomes
5-ASA/SSZ	2.4–4 g/day	Jung et al. (2012)	143/292	Retrospective cohort study	Positive effect in maintaining remission

THD	2-3 mg/kg/day	Yasui et al. (2008)	7	Case series	Dramatic improvement in clinical symptoms
	—	Lee et al. (2010)	4	Case series	3/4 patients had a clinical improvement and all discontinued steroid therapy

THD/IFX/ADA/ ETA	THD 50–100 mg/day IFX 5 mg/kg every 8 weeks ADA 160 mg at week 0 and 80 mg at week 2, followed by 40 mg every other week ETA 25 mg twice a week	Hatemi et al. (2015)	13/64	Observational study	Remission obtained with TNF-*α* antagonists and/or THD in about 75% of cases.

AZA or 6-MP	AZA 2–2.5 mg/kg/day 6-MP 0.5–1.5 mg/kg/day	Jung et al. (2012)	67/272	Retrospective study	Relative good effect for maintenance of remission

AZA or 6-MP vs 5-ASA	AZA 2–2.5 mg/kg/day or 6-MP 1–1.5 mg/kg/day vs 5-ASA 3-4 g/day	Lee et al. (2015)	77	Retrospective observational study	The rates of reoperation, readmission, and death were not significantly different between the 5-ASA and thiopurine groups

MTX + IFX	MTX - FX 3–5 mg/kg every 8 weeks	Iwata et al. (2011)	10	Observational study	Long-term alleviation of entero-BD and excellent tolerability with combination of IFX and MTX

INF-*α*	6 × 10^6^ IU per day for 14 days	Grimbacher et al. (1997)	1	Case report	Complete remission of Behçet's retinal infiltrates and BD-related colitis
	3 × 10^6^ IU/day 3 times/week increased to 6 × 10^6^ IU/day 3 times/week	Monastirli et al. (2010)	1	Case report	Complete remission of all clinical manifestations

IVIg	400 mg/kg/day for 5 days per month	Cantarini et al. (2016)	1/4	Case series	Complete disease remission of gastrointestinal, manifestations

IFX	—	Ideguchi et al. (2014)	7/43	Retrospective observational study	Good response in two patients, remission in one, partial response in two, and unchanged GI lesions in two patients
	5 mg/kg/every 8 weeks	Lee et al. (2013)	28	Multicenter retrospective study	IFX efficacy for patients with moderate-to-severe intestinal BD
	5 mg/kg every 8 weeks	Kinoshita et al. (2013)	15/43	Retrospective cohort study	Acceptable efficacy of IFX in BD patients refractory to conventional treatments
	5 mg/kg every 8 weeks	Hibi et al. (2016)	11/18	Open-label study	IFX efficacy in the treatment of intestinal BD

ADA	160 mg at week 0 and 80 mg at week 2, followed by 40 mg every other week	Tanida et al. (2015)	20	Multicenter, open-label, uncontrolled study	ADA effectiveness in inducing and maintaining clinical improvement and remission in patients with intestinal BD
	160 mg at week 0 and 80 mg at week 2, followed by 40 mg every other week	Tanida et al. (2016)	8	Retrospective observational study	Long-term efficacy and safety of ADA for the treatment of intestinal BD in the clinical setting

ETA	25 mg twice a week	Ma et al. (2014)	19/35	Observational study	The relapse rate for etanercept therapy was reduced significantly when compared with conventional therapy

ANA	100 mg/day 2 mg/kg/day increased to 2.5 mg/kg/day	Cantarini et al. (2013)	3/9	Case series	Complete resolution of abdominal pain in two patients, relapse in one patient

CANA	150 mg every 8 weeks 150 mg every six weeks	Vitale et al. (2013)	2/3	Case series	Complete resolution of abdominal pain

TCZ	8 mg/kg/ every 4 weeks	Deroux et al. (2015)	3/4	Case series	Less effective for arthralgia and abdominal pain

ADA, adalimumab; ANA, anakinra; anti-TNF-*α*, anti-tumor necrosis factor-*α*; 5-ASA, 5-aminosalicylic acid; AZA, azathioprine; CANA, canakinumab; ETA, etanercept; INF-*α*, interferon *α*; IFX, infliximab; IVIg, intravenous immunoglobulins; 6-MP, 6-mercaptopurine; MTX, methotrexate; SSZ, sulfasalazine; TCZ, tocilizumab; THD, Thalidomide.
